# Language Familiarity and Proficiency Leads to Differential Cortical Processing During Translation Between Distantly Related Languages

**DOI:** 10.3389/fnhum.2021.593108

**Published:** 2021-02-26

**Authors:** Katsumasa Shinozuka, Kiyomitsu Niioka, Tatsuya Tokuda, Yasushi Kyutoku, Koki Okuno, Tomoki Takahashi, Ippeita Dan

**Affiliations:** Research and Development Initiatives, Applied Cognitive Neuroscience Laboratory, Chuo University, Tokyo, Japan

**Keywords:** word translation, foreign language, linguistic distance, English proficiency, word familiarity, functional near infrared spectroscopy (fNIRS), brain activation

## Abstract

In the midst of globalization, English is regarded as an international language, or Lingua Franca, but learning it as a second language (L2) remains still difficult to speakers of other languages. This is true especially for the speakers of languages distantly related to English such as Japanese. In this sense, exploring neural basis for translation between the first language (L1) and L2 is of great interest. There have been relatively many previous researches revealing brain activation patterns during translations between L1 and English as L2. These studies, which focused on language translation with close or moderate linguistic distance (LD), have suggested that the Broca area (BA 44/45) and the dorsolateral prefrontal cortex (DLPFC; BA 46) may play an important role on translation. However, the neural mechanism of language translation between Japanese and English, having large LD, has not been clarified. Thus, we used functional near infrared spectroscopy (fNIRS) to investigate the brain activation patterns during word translation between Japanese and English. We also assessed the effects of translation directions and word familiarity. All participants’ first language was Japanese and they were learning English. Their English proficiency was advanced or elementary. We selected English and Japanese words as stimuli based on the familiarity for Japanese people. Our results showed that the brain activation patterns during word translation largely differed depending on their English proficiency. The advanced group elicited greater activation on the left prefrontal cortex around the Broca’s area while translating words with low familiarity, but no activation was observed while translating words with high familiarity. On the other hand, the elementary group evoked greater activation on the left temporal area including the superior temporal gyrus (STG) irrespective of the word familiarity. These results suggested that different cognitive process could be involved in word translation corresponding to English proficiency in Japanese learners of English. These difference on the brain activation patterns between the advanced and elementary group may reflect the difference on the cognitive loads depending on the levels of automatization in one’s language processing.

## Introduction

In the midst of globalization, English is regarded as an international language, or Lingua Franca (Seidlhofer, 2005; Crystal, 2012; Kirkpatrick, 2012), with the number of worldwide English speakers being over 2 billion (Crystal, 2008). However, it is evident that the English proficiency of Japanese learners is fairly low regarding English test scores including the Test of English for International Communication (TOEIC; Educational Testing and Service, 2020b), the International English Language Testing System (IELTS; IELTS Partners, 2020), and the Test of English as a Foreign Language (TOEFL; Educational Testing and Service, 2020a) in comparison to English learners in other nations.

Difficulty in handling English (second language: L2) for Japanese may be associated with linguistic reasons. The Japanese language (first language: L1) is the most distant from English in terms of linguistic distance (LD), which is mainly based on morphological, phonological, and syntactic elements (Chiswick and Miller, 2005). LD to English ranges from the lowest score (hardest to learn) of 1.00 for Japanese to the highest score (easiest to learn) of 3.00 for Afrikaans, Norwegian, and Swedish. The LD score is determined by the ease/difficulty that Americans have learning different foreign languages, and it corresponds fairly well with differences in foreigners’ ease/difficulty in learning English. For L1 speakers to process a L2 with a large LD is not an easy process. However, little is known about cognitive aspects of processing a distantly related L2. One possible approach may be to understand the neural basis for L2 handling by linguistically distant L1 speakers. In particular, we focused on exploring the neural basis of translation because it is an indispensable part of L1 speakers’ handling of L2. Before exploring specific aspects of L2 with a large LD, we will first introduce existing models of the word product (output) system underlying translation for L2 speakers in general. We will then review important behavioral and neuroscience experiments on translation conducted for L2 speakers in general. Finally, we will interpret the results of these experiments from a cognitive processing perspective.

The bilingual lexico-semantic system is an analytical cognitive model of L2 speakers’ second language acquisition of words themselves and their meanings (Votaw, 1992). The system consists of several distinct elements: how the word looks (orthography), how it sounds (phonology), what it means (semantics), what syntactic properties it has (lemmas), and how it is pronounced (an output system that specifies the pronunciation of word forms) (Patterson et al., 1987; Indefrey and Levelt, 2000; Meyer et al., 2016). The bilingual lexico-semantic system is known to support a variety of linguistic activities such as reading, speaking, and switching between languages in translation in other (second) languages (Votaw, 1992; Price et al., 1999). Particularly, word translation by L2 speakers requires the speaker to generate the translation equivalent of the presented word rather than to merely name it (Green, 1986). In addition, these cognitive operations are assumed to be accomplished by modulating the activation of the language system (Grosjean, 1997; Paradis, 1997) with the inhibition control system, which is described under the scheme of the inhibition control (IC) model (Green, 1998; Ong and Zhang, 2010). This model sets and maintains the target, avoids naming words in L1, and instead produces the equivalent translation as a response. Therefore, it is assumed that the bilingual lexico-semantic system works accurately when the inhibition control system (Green, 1998; Ong and Zhang, 2010) adequately controls language processing.

Moreover, psycholinguistic data emphasize two different routes for translation (Kroll and Stewart, 1994; Kroll and De Groot, 2002; Duyck and Brysbaert, 2008): a non-semantic direct route (lexical route) in which the word forms of translation equivalents are linked at the lemma level (Jescheniak and Levelt, 1994) and an indirect route (semantic route) in which they are connected via their meaning (i.e., their lexical concepts). According to the IC model, word selection along either route involves lemma activation and the inhibition of lemmas with a non-target language tag. The involvement of these two routes is thought to differ depending on the direction of word translation (L1-into-L2 or L2-into-L1) (Jescheniak and Levelt, 1994; Price et al., 1999). In L1-into-L2 translation the semantic route is dominant, whereas in L2-into-L1 translation the lexical route is dominant, reflecting the acquisition of the L2 word in the context of a pre-existing lexical concept-word form link in L1 (Price et al., 1999). In fact, Kroll and Stewart (1994) suggested through experimental studies that L1-into-L2 translation may produce more semantic processing than L2-into-L1 translation does. Thus, it is of great importance to explore the neural basis of translation by examining cortical activation patterns in both directions, L1-into-L2 and L2-into-L1.

There are some behavioral experiments using word translation tasks. De Groot and Poot (1997) examined the performance of balanced bilinguals, translating one set of words from L1, Dutch, to L2, English, and vice versa. The LD between Dutch and English is known to be close, scored as 2.75 (Chiswick and Miller, 2005). Reaction time for word-translation of L1 into L2 was longer than that of L2 into L1 and there were high error rates while translating L1 into L2. Kroll et al. (2010) also conducted a similar experiment in which balanced bilinguals translated simple L1 (English) sentences into L2 (French with a LD of 2.5) and vice versa (Chiswick and Miller, 2005). Their results were mostly in line with those by De Groot and Poot (1997), replicating more prolonged reaction time and higher error rate while translating L1 into L2 than L2 into L1. From these behavioral studies, it could be said that translation from L1 into L2 is cognitively more loaded than that from L2 into L1. Moreover, considering that these experiments were conducted for balanced bilinguals, it is also suggested that the mental lexicon in L2 may be smaller than that in L1 regardless of bilingualism levels (De Groot and Poot, 1997; Kroll et al., 2010).

With advancements in functional brain imaging, many studies have started to focus on brain activation patterns during translations between L1 and L2. Many of these studies recruited balanced bilinguals and examined brain activities during translation between languages with close LDs. Most studies performed thus far used PET (positron emission tomography), which is invasive in terms of the intake of radioactive substances, but is relatively unrestrictive regarding body motion and language—related behaviors, and thus is suitable for functional neuroimaging during translation. On the other hand, probably due to technical constraints, fMRI (functional magnetic resonance imaging) has not yet been applied directly for neuroimaging examination of bidirectional translation between L1 and L2, to our knowledge. Rather, fMRI has been used to reveal the cognitive mechanisms behind more fundamental processes of translation, such as the learning process of unknown L2 words (Mayer et al., 2015) and judging the correctness of translated texts (Lehtonen et al., 2005). Fortunately, Hervais-Adelman et al. (2015) examined the neural basis of translation with a focus on language translation from L1 to L2 only. They aimed to clarify how multilinguals who had a high level of language proficiency in at least three languages exhibited brain activation during simultaneous interpretation of L1 (their most fluent language: English or French) to L2 (9 target languages such as French, Spanish, Italian, and German). As a result, they confirmed the involvement in the translation of the anterior portion of Broca’s area (BA 45). This finding cannot be discussed from a LD-based perspective (Chiswick and Miller, 2005) because participants did not necessarily translate English as the L2, but it is important in clarifying the neural basis of translation. There are also studies showing that the functional connectivity of the brain is different between L1-into-L2 and L2-into-L1 translation (Zheng et al., 2020). Zheng et al. (2020) demonstrated that functional connectivity between a core semantic hub (the left anterior temporal lobe, ATL) and key nodes of attentional and vigilance networks (left inferior frontal, left orbitofrontal, and bilateral parietal clusters) increased during L1-into-L2 translation, whereas functional connectivity was observed only between the left ATL and the right thalamus, regions implicated in the automatic relaying of sensory information to cortical regions, during L2-into-L1 translation. These results may imply that enhanced functional connectivity between semantic and attentional mechanisms is involved during L1-into-L2 translation (Zheng et al., 2020). The finding in Zheng et al. (2020) is consistent with the assumption in the IC model that two different routes are involved depending on the direction of word translation (L1-into-L2 or L2-into-L1) (Jescheniak and Levelt, 1994; Price et al., 1999).

Some PET studies have examined brain activation during bidirectional language translation between L1 and L2 directly, and we will review them in detail here. Klein et al. (1995) used PET to investigate brain activation patterns during a word translation task between French and English with a close LD of 2.50 (Chiswick and Miller, 2005). Participants whose L1 was English but were also proficient in French (L2) translated L1 into L2 and vice versa. While translating L1 into L2, the left frontal ventrolateral cortex (BA 10/47), the left dorsolateral cortex (BA 8), the left temporal inferotemporal cortex (BA 37/20), the left parietal cortex (BA 7), and the cerebellum (Vermis) were activated. While translating L2 into L1, the left frontal ventrolateral cortex (BA 10/47; BA 9/46), the left dorsolateral cortex (BA 8), the left temporal inferotemporal cortex (BA 37/20), the left parietal cortex (BA 7), the cerebellum (right), and the thalamus/pulvinar were activated. Price et al. (1999) examined brain activities during translation between German (L1) and English (L2), having a close LD of 2.25 (Chiswick and Miller, 2005), on balanced bilinguals using PET. While translating both L1 words into L2 and vice versa, the left anterior cingulate, the left supplementary motor area and the left medial fusiform, the bilateral subcortical structures, the anterior insula, and the cerebellum were activated. Quaresima et al. (2002) examined brain activation while balanced bilinguals of Dutch (L1) and English (L2), with a close LD of 2.75 (Chiswick and Miller, 2005), translated easy sentences from L1 into L2 and vice versa, using fNIRS (functional near infrared spectroscopy), which offers non-invasive hemodynamic assessment in a natural environment, and thus is useful for this purpose. Among the lateral frontal and temporal regions covered in the fNIRS measurement, the left cortical area surrounding Broca’s area (BA 44/45) was activated irrespective of translation direction.

In addition, there are a few studies focusing on brain functions during translation for English learners whose L1 is moderately distant from English. In a PET study, Rinne et al. (2000) examined brain activation of professional interpreters during translation from Finnish (L1) to English (L2) having a moderately close LD score of 2.0 (Chiswick and Miller, 2005). Activation patterns were asymmetric as to direction of translation. While translating L2 into L1, activations of the left ventrolateral frontal cortex (BA 46), and the left premotor cortex (BA 6) were observed. On the other hand, while translating L1 into L2, the left ventrolateral frontal cortex (BA 45), the left: inferior temporal cortex (BA 20/28), the left premotor cortex (BA 6), and the cerebellum were activated.

To summarize the major functional neuroimaging studies on translation presented above, various regions were activated while translating from L1 into L2 and vice versa. Moreover, the brain activation patterns were different depending on translation direction. Though there were different activation patterns during translation across studies, the area surrounding the left prefrontal cortex, such as the left ventrolateral frontal cortex involved in Broca’s area and the left dorsolateral prefrontal cortex (DLPFC), was activated consistently. This was applicable to the studies focusing on language translation with close LDs (Klein et al., 1995; Price et al., 1999; Quaresima et al., 2002) but also to those with moderate LDs (Rinne et al., 2000). Broca’s area, in particular, has been reported to be active regardless of the direction of translation (L1-into-L2 and L2-into-L1) in a study focusing on translation from both directions (Quaresima et al., 2002). This region is responsible for retrieving linguistic information (Klein et al., 1995) and is also related to verbal working memory (Paulesu et al., 1993), morphosyntactic processing (Laine et al., 1999), and semantic analysis (Cabeza and Nyberg, 1997). The left DLPFC plays an important role for working memory associated with translation (Klein et al., 1995) and language encoding and semantic processing (Rinne et al., 2000). These frontal regions are more widely activated during L1-into-L2 translation (Rinne et al., 2000). In addition, the left inferior temporal activation was observed in Klein et al. (1995) and Rinne et al. (2000). This region belongs to the so-called ‘basal temporal language area’ which has been related to word-finding (Lüders et al., 1991; Damasio et al., 1996) and semantic processing (Vandenberghe et al., 1996; Seghier and Price, 2012). The function of these temporal regions during language translation is thought to be primarily responsible for the semantic processing of language (Klein et al., 1995; Rinne et al., 2000).

The functional meaning of these brain regions is consistent with the mental representational model of second language acquisition. That is, these areas are involved in both word production and word perception (Lüders et al., 1991; Indefrey and Levelt, 2000; Indefrey and Levelt, 2004; Hamberger and Cole, 2011), and are therefore likely to be active in common even between languages with a close or moderate LD. On the other hand, the widespread activation including the temporal region during L1-into-L2 translation may reflect the dominance of the semantic route (Jescheniak and Levelt, 1994; Price et al., 1997). Thus, it is likely that the left prefrontal cortex and surrounding area are the regions generally involved in language translation, and that other regions might be differentially recruited depending on differences in LD and on the direction of translation.

Although these findings provided valuable insights into understanding the cognitive processes underlying L2 handling, there are limitations to applying them to understanding cognitive processes of Japanese speakers handling English, a most distantly related language with a LD of 1.0. First, previous studies have mainly been conducted on balanced bilinguals who could effortlessly translate L1 into L2 and vice versa. Because their performance is not expected to be similar to Japanese learners of English, whether brain activation patterns observed in previous studies are also applicable to the language translation process of Japanese learners or not is unclear. Second, those previous studies focused on language translation between English and other languages whose LD is close or moderate. The LD between Japanese and English is the most distant along with that between Korean and English (Chiswick and Miller, 2005). In fact, it has been shown that differences in LD produce different patterns of brain activation during language processing, such as sentence comprehension (Jeong et al., 2007). Accordingly, the results of previous studies might not be directly adapted to translation between Japanese and English.

Therefore, in the current study, we aimed to investigate brain activation patterns while Japanese learners of English translated Japanese words into English and vice versa. In so doing, we have to take the following issues into consideration. First, the large LD, literally entailing difficulty in L2 learning, leads to the emergence of various levels of Japanese learners of English. Since the level of English acquisition may affect the brain activation patterns during translation, we examined both advanced Japanese learners of English who might easily translate L1 into L2 and vice versa and elementary learners who might not easily do the same thing. Second, it is often too difficult for elementary-level English learners to translate Japanese sentences into English and vice versa. Thus, we adopted word translation as vocabulary knowledge is indispensable for acquiring L2 and allows the measurement of individual English skills (Laufer and Nation, 1999). Third, we have to consider the familiarity issue. When adopting L1 and L2 words as stimuli, it might be difficult to distinguish whether the observed cognitive reactions are attributed to qualitative differences of languages or to quantitative differences of cognitive loads. Thus, in order to examine the effects of word familiarity, we adopted high— and low-familiarity L1 and L2 words as stimuli.

Language translation is a linguistic activity that is commonly practiced on a daily basis in an environment where a second language is used. Thus, it is desirable to measure brain activations while translating in a less-restrictive environment that is as close as possible to normal daily life. Although most previous studies used PET and a large body of linguistic studies used fMRI, their experimental environments presented a rather restricted and unfamiliar environment in which participants performed translation. However, fNIRS can measure brain activation patterns by simply placing probes on the head under conditions close to everyday life, such as participants having freedom of movement, and was proven to be useful in a pioneering study by Quaresima et al. (2002) on translation. fNIRS has been successfully adopted in other language-related studies including language acquisition (Obrig et al., 2010, 2017; Homae et al., 2011; May et al., 2018; Sugiura et al., 2018), speech perception (Minagawa-Kawai et al., 2002; Minagawa-Kawai et al., 2004; Minagawa-Kawai et al., 2007), and speech comprehension (Lei et al., 2018). Hence, we used fNIRS to measure brain activations during translation of Japanese (L1) and English (L2) words, taking into consideration language direction and word familiarity as within subject factors in both high- and low-proficiency English learners.

## Materials and Methods

### Participants

Forty-three healthy right-handed Japanese young adults (23 males and 20 females, mean age 20.81 ± 1.37, age range 18 – 25) participated in this study. All participants had taken the TOEIC^®^ Listening and Reading test within the past year. TOEIC^®^ is the most widely used standardized examination with a yearly participation rate of over two million and its sufficiency in reliability and validity has been reported by Lawson (2008). Participants who received a score of over 730 points were assigned to the advanced group and participants who received a score below 470 points were assigned to the elementary group based on the TOEIC^®^ official standard (Educational Testing and Service, 2020b). This official standard indicates that those who scored 730 points or more “have the ability to communicate appropriately in any situation” or “can communicate adequately at a similar level to a native speaker,” while those who scored 470 or less “have a minimum level of communication in a daily conversation” or “cannot communicate at all.”

Among the initial 43 participants, three were excluded from the data analysis. One misunderstood the instructions. Another was excluded due to instrumental trouble during the fNIRS experiment, and the third was recognized as left-handed, based on the Edinburgh inventory (Oldfield, 1971). The remaining participants consisted of 21 in the advanced-level group and 19 in the elementary-level group. Participants’ average score in the advanced group was 826.36 ± 67.93 (max: 975, min: 740) and that in the elementary group was 377.50 ± 69.80 (max: 460, min: 225).

The experimental protocols were approved by the Institutional Review Board (IRB) of Chuo University and it was in accordance with the Declaration of Helsinki guidelines. written informed consent was obtained from all participants in advance.

### Stimuli and Experimental Design

**FIGURE 1:**
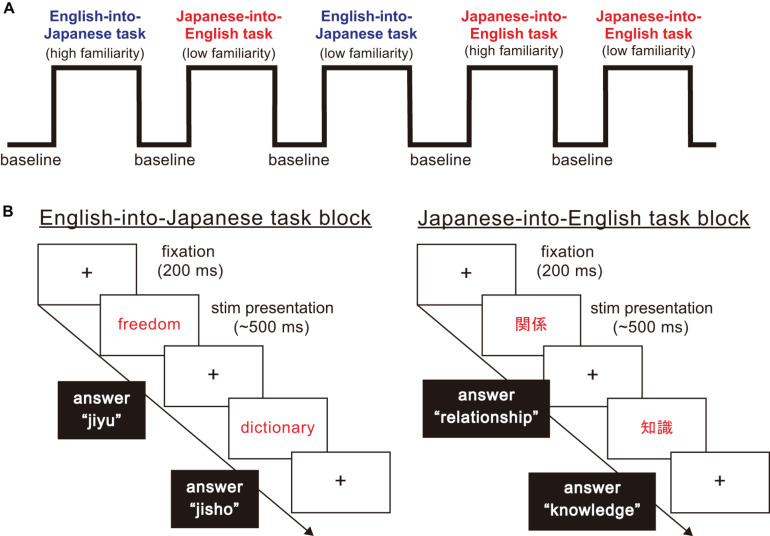
The structure of the word translation task paradigm. **(A)** The word translation task paradigm consisted of baseline and task blocks. There were four types of task blocks arising from combinations of translation direction (English into Japanese/Japanese into English), and high and low familiarity. **(B)** For each task block, a fixation point was presented for 200 ms. Then, a stimulus word shown in English or Japanese was presented in the center of the display. When English words were presented, participants were asked to translate and type corresponding Japanese words in Roman letters. When Japanese words were presented, participants were asked to translate and type corresponding English words.

In this study, participants were asked to perform a word translation task between Japanese and English as quickly as possible. As Figure 1 shows, the stimuli in this experiment were divided into three task blocks, namely non-translation as baseline blocks, English-into-Japanese task blocks and Japanese-into-English task blocks. There were four task conditions in the task blocks: translation direction (English-into-Japanese/Japanese-into-English) × familiarity (high/low familiarity). All participants were required to answer by typing the spelling of the words in English or in Japanese using Roman letters on a keyboard. Japanese people habitually use Roman letters when typing Japanese words. For this reason, we decided that the balance of control was not affected between typing English letters and Japanese Roman letters. In baseline blocks, they were asked to transcribe Japanese words written in black into Roman letters without translating into Japanese or English (e.g., “平成” to “heisei” in Roman letters). In Japanese-into-English task blocks, they were asked to translate Japanese words written in red into corresponding English words and to type them [e.g., “車” in Japanese Kanji character(s) to car in English]. In English-into-Japanese task blocks, they were asked to translate English words written in red in Roman letters into corresponding Japanese words and to type them in Roman letters (e.g., “world” to “sekai” in Roman letters). In all the task blocks, the participants were asked to press the “SPACE” bar immediately after they produced the translated or control word in their mind, type it on the keyboard, and finally press the “ENTER” key immediately after typing the translated words. If the participants did not produce the translated word, the next trial stimulus appeared on the computer monitor in five seconds. In baseline blocks, the times of the stimuli presentation were randomized, with the words appearing four or five times on a computer monitor, to avoid prediction of the timing of the subsequent trial. The number of stimuli presentation were three times for task blocks. The inter stimuli interval lasted 2 s.

Response time and the accuracy of the response were obtained while the participants conducted word translation. Concerning seemingly correct answers, we defined typing errors of two or more letters in a single word to be incorrect, but with one letter to be correct (e.g., mistyping “money” as “mooney” would be considered correct). We judged whether the answers included typing errors with independent visual examinations by three raters (KN, KO, and ToT). For stimuli presentation and response recording, we used the Psychoplrysics Toolbox (Brainard, 1997; Pelli, 1997; Kleiner et al., 2007), which operated in a Matlab (Mathworks, Natick, MA, United States) environment. The response for word translation task on the computer was synchronized temporally with fNIRS records through a serial port to record hemodynamic responses. Specific Japanese and English word stimuli were selected based on the following considerations. First, we set the word stimuli to comprise of only nouns because verbs, adjectives or other parts of speech tend to be polysemic, possibly making participants confused in grasping the meanings of the presented words. Second, we set word stimuli to be presented visually with Kanji, or Chinese characters, based on consultation with two professional simultaneous interpreters suggesting that Japanese words have many homonyms and cause higher chances of confusion when auditorily presented. In accordance, English words were also presented visually.

Basically, the stimuli in this study were chosen on a word familiarity basis both for Japanese and English words. This is because the most frequently used British National Corpus (BNC) was established based on English word frequency created by British English speakers, which was not suitable as word translation stimuli for Japanese (British National, and Corpus., 2007). Thus, we utilized the NTT Psycholinguistic Databases “Lexical Properties of Japanese” for the Japanese stimuli (Amano and Kondo, 1998) and English words familiarity ratings among Japanese for English stimuli (Yokokawa et al., 2007). Both corpora were based on familiarity ratings for English and Japanese words, respectively, for Japanese people. Word familiarity in both English and Japanese ranges from 1.0 to 7.0, with 7.0 being the most familiar, and 1.0 being the least.

Further, we utilized three English Japanese Dictionaries, namely the online Cambridge dictionary (Cambridge University, and Press., 2020), the OLEX English-Japanese Dictionary (Nomura et al., 2016) and the Genius English–Japanese Dictionary (Konishi and Minamide, 2001) to confirm whether the primary meaning of each selected noun was the same across the three dictionaries. In addition, we arranged visually presented Japanese words in Kanji, or Chinese characters, when necessary, to be included in the specific set of basic Kanji, “Joyo-Kanji,” which consists of 2135 characters intended for daily use (Agency for Cultural Affairs, 2010). For English words, the number of syllables was set from one to three. The mora of Japanese words was set from two to six. This was to enable participants to answer the questions (they were asked to translate Japanese/English words and type the spelling) within the limited time. We regarded two morae to be equivalent to one syllable as per Kubozono (1989).

Finally, for selecting high and low familiarity words both in Japanese as L1 and English as L2, we generated composite familiarity scores by adding the familiarity scores from the two corpora (Amano and Kondo, 1998; Yokokawa et al., 2007). Accordingly, 92 words with the highest and lowest scores, were selected as high and low familiarity words, respectively. Each averaged familiarity was 6.19 for high-familiarity words and 4.40 for low-familiarity words. They were significantly different in familiarity [*t*(182) = 41.93, *p* < 0.01, *d* = 3.11]. In addition, we selected 147 relatively common Japanese words as baseline words from Amano and Kondo (1998). Combinations of Kanji with Katakana or Hiragana characters (e.g., “子育て”; parenting, “銅メダル”; bronze medal) were excluded from these baseline word sets. All baseline words were written in Kanji characters like the task words. The averaged word familiarity was 6.02. There were no stimuli words which overlapped between baseline and task words.

### Data Acquisition

During the word translation task, we recorded hemodynamic responses using fNIRS measurement. We used a 52-channel continuous wave system (ETG-4000, Hitachi, Japan). Optical data from individual channels were collected at two different wavelengths, 695 and 830 nm, and analyzed using the modified Beer–Lambert Law (Delpy et al., 1988). Changes in the oxygenated hemoglobin (oxyHb) and deoxygenated hemoglobin (deoxyHb) signals were calculated in units of millimolar × millimeter (mM × mm) (Maki et al., 1995). The sampling rate was set to 10 Hz.

**FIGURE 2:**
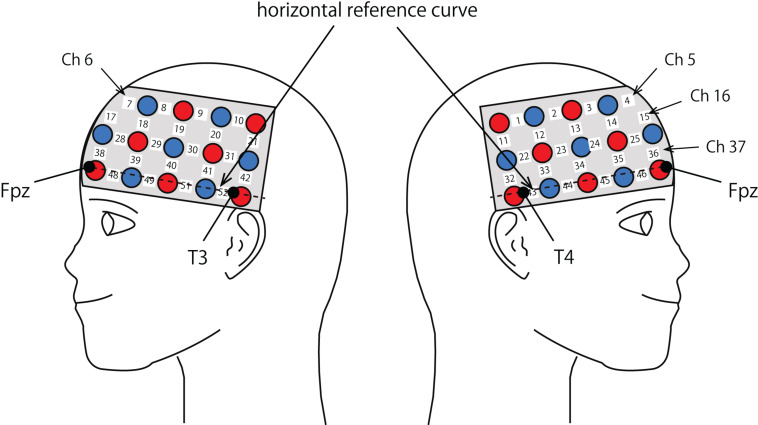
Spatial profiles of fNIRS channels. Left and right sides views of the probe arrangements are exhibited with fNIRS channel orientation. Detectors are indicated with blue circles, illuminators with red circles, and channels with white squares. Corresponding channel numbers are shown in black. Chs 5, 6, 16, and 37 are not visible, but located around or over the midline.

The probe was fixed using one 9 × 34 cm rubber shell over the frontal and temporal areas (Figure 2) in reference to previous studies (Niioka et al., 2018; Kawabata Duncan et al., 2019). The shell of 33 probes, consisting of a 3 × 11 array with 17 emitters and 16 detectors, allowed us to measure the relative concentration of hemoglobin at 52-channels. We defined the midpoint of a pair of illuminating and detecting probes as a channel location. We defined channel locations in accordance with the international 10–20 system for EEG (Klem et al., 1999; Jurcak et al., 2007). The fNIRS probes were placed such that Fpz coincided with the sixth probe in the middle column of holders in the 3 × 11 probe holder and the lower line substantially matched the horizontal reference curve, where the horizontal reference curve was determined by a straight line connecting FPz—T3—T4 (Jurcak et al., 2007). The inter-optode distance was 3 cm. For spatial profiling of fNIRS data, we adopted the probabilistic registration method (Okamoto and Dan, 2005; Singh et al., 2005; Tsuzuki et al., 2007; Tsuzuki and Dan, 2014) to register fNIRS data to Montreal Neurological Institute (MNI) standard brain space, which further allows us to estimate macroanatomical locations of the channels (Rorden and Brett, 2000).

### fNIRS Data Analysis

We used Matlab 2007b (The Mathworks, Inc., Natick, MA, United States) for fNIRS data analysis with several in-house toolboxes to realize the procedures to be described hereafter. Since the oxyHb signal is the most sensitive indicator of regional cerebral hemodynamic response (Huppert et al., 2006; Homae et al., 2007; Cui et al., 2010), we analyzed oxyHb signal changes. Individual timeline data for the oxyHb signal of each channel were preprocessed in the following way. First, we moving-averaged raw data for 5 s. Then, channels with a signal variation of 10% or less were considered defective measurements and excluded from analysis. To remove the influence of measurement noise such as breathing, cardiac movement and so on from the remaining channels, we applied wavelet minimum description length (Wavelet-MDL) (Jang et al., 2009).

After pre-processing oxyHb timeline data for each individual on each channel, we conducted General Liner Model (GLM) analysis with regression to hemodynamic response function (HRF). The regressors were created by convolving (Equation 2) the boxcar function N (t_*p*_,t) with the HRF shown in Equation 1 (Friston et al., 1998).

(1) $$h\,\left(\tau_{p},t\right)=\frac{t^{\tau_{p}}\,e^{-t}}{\left(\tau_{p}\right)!}-\frac{t^{\tau_{p}+\tau_{d}}\,e^{-t}}{A\,\left(\tau_{p}+\tau_{d}\right)!},$$

(2) $$\text{f}\,\left(\tau_{\text{p}},t\right)=h\,\left(\tau_{\text{p}},t\right)*N.$$

**FIGURE 3:**
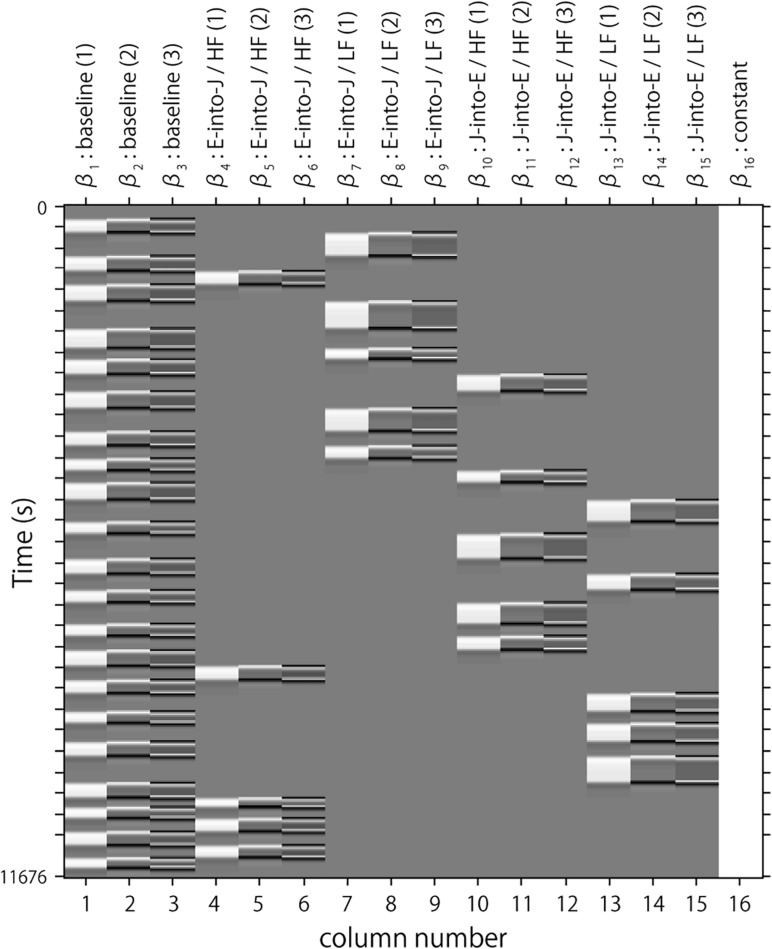
An example of a design matrix, X. The row indicates time from up to bottom. The first to third columns indicate the canonical HRF (hemodynamic response function), and the first and second derivatives, respectively, for baseline trials. The fourth to sixth columns indicate the canonical HRF, and the first and second derivatives, respectively, for task trials (English into Japanese/high familiarity). The seventh to ninth columns indicate the canonical HRF, and the first and second derivatives, respectively, for task trials (English into Japanese/low familiarity). The 10th to 12th columns indicate the canonical HRF, and the first and second derivatives, respectively, for task trials (Japanese into English/high familiarity). The 13th to 15th columns indicate the canonical HRF, and the first and second derivatives, respectively, for task trials (Japanese into English/low familiarity). The 16th column indicates the constant.

Following the conventional usage, we set the first peak delay, t_*p*_, to 6 s, the second peak delay, t_*d*_, to 10 s, and A, the amplitude ratio between the first and second peak, to 6 s. The first and second derivatives were included to reduce the influence of noise of individual data further. The specific design matrix is shown in Figure 3. Columns 1, 2, and 3 in Figure 3 respectively represent the HRF of the baseline block and its first and second derivatives. Columns 4, 5, and 6 respectively represent the HRF of the English-into-Japanese/high-familiarity task block and its first and second derivatives. Columns 7, 8, and 9 respectively represent the HRF of the English-into-Japanese/low-familiarity task block and its first and second derivatives. Columns 10, 11, and 12 respectively represent the HRF of the Japanese-into-English/high-familiarity task block and its first and second derivatives. Columns 13, 14, and 15 respectively represent the HRF of the Japanese-into-English/low-familiarity task block and its first and second derivatives. Column 16 represents the constant.

We used the β value as an indicator of the oxyHb signal for each regressor. Among 16 β values, the four β values (β_4_, β_7_, β_10_, β_13_) representing the task block served for further statistical analyses, while the others were regressed out. β_4_ was the indicator of the brain activity during the task period English-into-Japanese/high familiarity and β_7_ is the indicator of the brain activity during the task period English-into-Japanese/low familiarity. Similarly, β_10_ is the indicator of the brain activity during the task period Japanese-into-English/high familiarity and β_13_ is the indicator of the brain activity during the task period Japanese-into-English/low familiarity. A one-sample *t*-test against zero was performed on β values for each task block and channel at the group level. Family wise errors for the *p*-values were corrected using Bonferroni correction. With the Bonferroni method, the statistical significance level (*a*) is divided by the number of channels, resulting in it being too conservative. The present study is the first to focus on Japanese–English translation, which has a large LD, entailing difficulty in L2 learning, and it was necessary to avoid the type II errors of missing the channels that were truly activated. Therefore, we will discuss “activated channels” based on sufficient effect sizes being obtained not only for significant channels (*a* = 0.05), but also for marginally significant channels (*a* = 0.10).

Further, we conducted a three-way mixed analysis of variance (ANOVA) with group (advanced/elementary) as the between subject factor and direction (English-into-Japanese/Japanese-into-English) and familiarity (high/low) as the within-subject factors on β values for each task block. β values were averaged between channels corresponding to the same anatomical label for channel activated in a one-sample *t*-test against zero. A simple main effect test was performed when an interaction between factors was significant. Statistical significance was set *a priori* at *p* < 0.05 for all comparisons.

### Behavior Data Analysis

We used IBM SPSS Statistics 25 for behavior data analyses. First, we averaged reaction time and accuracy at the individual level for each of the four task blocks. Then, at the group level, we conducted a three-way mixed ANOVA with group (advanced/elementary), direction (English-into-Japanese/Japanese-into-English), and familiarity (high/low) as the within-subject factors on RTs and accuracy for each task block. A simple main effect test was performed when an interaction between factors was significant. A two-way interaction contrast for each of group was tested to confirm how familiarity contrasts differ depending on translation direction (English-into-Japanese/Japanese-into-English) when a three-way interaction was significant. Thus, for each group, we first calculated the contrast between translation direction (English-into-Japanese minus Japanese-into-English) under each familiarity condition to generate two contrasts: English-into-Japanese minus Japanese-into-English contrast for high and low familiarity words, respectively. From these, we further generated a two-way interaction contrast for each group to represent the difference between high and low familiarity words, namely, [English-into-Japanese minus Japanese-into-English for high familiarity words] minus [English-into-Japanese minus Japanese-into-English for low familiarity words]. For each group, a one-sample *t*-test against zero was performed on the obtained contrast. Statistical significance was set *a priori* at *p* < 0.05 for all comparisons.

## Results

### fNIRS Data

**FIGURE 4:**
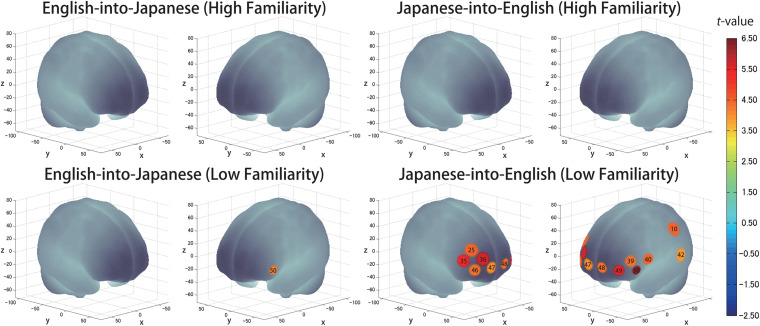
The results of the group analysis for the advanced group. Family wise errors due to multichannel measurement were corrected using the Bonferroni method. Significant *t*-values for MNI-registered channels are indicated by the color scale.

**FIGURE 5:**
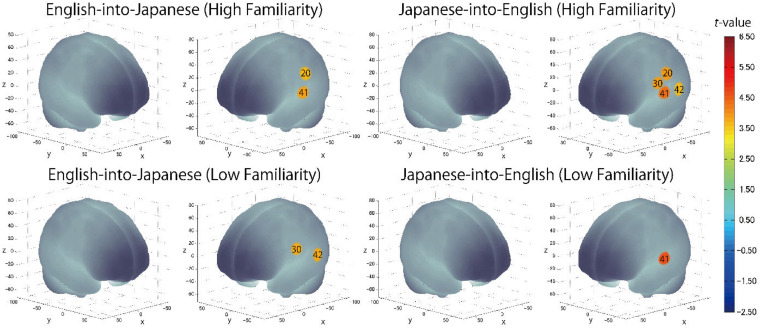
The results of the group analysis for the elementary group. Family wise errors due to multichannel measurement were corrected using the Bonferroni method. Significant *t*-values for MNI-registered channels are indicated by the color scale.

The results of the group analysis with a one-sample *t-*test showed that, for the advanced group, significant oxyHb signal increase was found in one channel for β_7_, the indicator of the canonical HRF for task trials (English-into-Japanese/low familiarity) [channel 50 (*t*(20) = 4.20, *p* < 0.05, *d* = 0.92] and in twelve channels for β_13_, the indicator of the canonical HRF for task trials (Japanese-into-English/low familiarity) [channel 10, *t*(20) = 4.48, *p* < 0.05, *d* = 0.98; channel 25, *t*(20) = 4.53, *p* < 0.05, *d* = 0.99; channel 35, *t*(20) = 5.32, *p* < 0.05, *d* = 1.16; channel 36, *t*(20) = 5.63, *p* < 0.05, *d* = 1.23; channel 39, *t*(20) = 4.34, *p* < 0.05, *d* = 0.95; channel 40, *t*(20) = 4.48, *p* < 0.05, *d* = 0.98; channel 42, *t*(20) = 3.88, *p* < 0.05, *d* = 0.85; channel 46, *t*(20) = 4.40, *p* < 0.05, *d* = 0.96; channel 47, *t*(20) = 4.11, *p* < 0.05, *d* = 0.90; channel 48, *t*(20) = 4.39, *p* < 0.05, *d* = 0.96; channel 49, *t*(20) = 5.47, *p* < 0.05, *d* = 1.19; and channel 50, *t*(20) = 6.36, *p* < 0.05, *d* = 1.39] when correcting multiplicity with the Bonferroni method (Figure 4 and Table 1). In contrast, the elementary group showed significant oxyHb signal increase in one channel for β_4_, the indicator of the canonical HRF for task trials (English-into-Japanese/high familiarity) [channel 41, *t*(18) = 3.94, *p* < 0.05, *d* = 0.86], in two channels for β_7_, the indicator of the canonical HRF for task trials (English-into-Japanese/low familiarity) [channel 30, *t*(18) = 3.77, *p* < 0.10, *d* = 0.82 and channel 42, *t*(18) = 3.75, *p* < 0.10, *d* = 0.82], in four channels for β_10_, the indicator of the canonical HRF for task trials (Japanese-into-English/high familiarity) [channel 20, *t*(18) = 3.96, *p* < 0.05, *d* = 0.87; channel 30, *t*(18) = 3.96, *p* < 0.05, *d* = 0.86; channel 41, *t*(18) = 4.34, *p* < 0.05, *d* = 0.95; and channel 42, *t*(18) = 3.65, *p* < 0.10, *d* = 0.80], and in one channel for β_13_, the indicator of the canonical HRF for task trials (Japanese-into-English/low familiarity) [channel 41, *t*(18) = 4.67, *p* < 0.01, *d* = 1.02] (Figure 5 and Table 2).

**TABLE 1:** Most likely estimated locations of activated channels from the probabilistic registration method in the advanced group.

Advanced group									

	*x*	*y*	*z*	*SD*	Anatomy	%	*t*	*p*	*d*
**English (L2) into Japanese (L1)/low familiarity**									
Ch 50	–54.3	38.3	–3.3	8.0	L-Broca’s area (BA 45)	71.5	4.20	0.0004	0.92
**Japanese (L1) into English (L2)/low familiarity**									
Ch 10	–62.0	–33.7	49.3	12.6	L-Wernicke’s area (BA 40)	63.2	4.48	0.0002	0.98
Ch 25	38.3	54.7	27.7	11.9	R-DLPFC (BA 46)	90.5	4.53	0.0002	0.99
Ch 35	50.0	49.3	12.3	10.8	R-DLPFC (BA 46)	67.1	5.32	<0.0001	1.16
Ch 36	27.7	68.3	14.3	11.5	FPA (BA 10)	95.5	5.63	<0.0001	1.23
Ch 39	–49.3	46.3	11.3	10.0	L-Broca’s area (BA 45)	54.9	4.34	0.0003	0.95
Ch 40	–59.7	19.3	11.3	10.3	L-Broca’s area (BA 44)	33.5	4.48	0.0002	0.98
Ch 42	–70.0	–40.7	8.7	12.0	L-STG (BA 22)	75.2	3.88	0.0009	0.85
Ch 46	40.3	63.7	–1.3	10.5	FPA (BA 10)	61.9	4.40	0.0003	0.96
Ch 47	14.3	73.0	–0.3	10.6	FPA (BA 10)	66.6	4.11	0.0005	0.90
Ch 48	–15.0	73.0	0.0	9.8	FPA (BA 10)	61.3	4.39	0.0003	0.96
Ch 49	–39.7	61.3	–2.0	9.2	FPA (BA 10)	65.1	5.47	<0.0001	1.19
Ch 50	–54.3	38.3	–3.3	8.0	L-Broca’s area (BA 45)	71.5	6.36	<0.0001	1.39

*SD indicates standard deviation in the spatial estimate; BA indicates Brodmann area; DLPFC indicates dorsolateral prefrontal cortex; FPA indicates frontopolar area; STG indicates superior temporal gyrus; L and R indicates left and right hemisphere.*

**TABLE 2:** Most likely estimated locations of activated channels from the probabilistic registration method in the elementary group.

Elementary group									

	*x*	*y*	*z*	*SD*	Anatomy	%	*t*	*p*	*d*
**English (L2) into Japanese (L1)/high familiarity**									
Ch 20	–66.0	–18.3	36.3	11.6	L-S1 (BA 2)	43.9	3.70	0.0007	0.81
Ch 41	–67.0	–11.7	8.7	11.5	L-STG (BA 22)	71.6	3.94	0.0004	0.86
**English (L2) into Japanese (L1)/low familiarity**									
Ch 30	–65.0	–0.3	24.7	10.5	L-Subcentral area (BA 43)	70.2	3.77	0.0007	0.82
Ch 42	–70.0	–40.7	8.7	12.0	L-STG (BA 22)	75.2	3.75	0.0007	0.82
**Japanese (L1) into English (L2)/high familiarity**									
Ch 20	–66.0	–18.3	36.3	11.6	L-S1 (BA 2)	43.9	3.96	0.0006	0.87
Ch 30	–65.0	–0.3	24.7	10.5	Subcentral area (BA 43)	70.2	3.96	0.0004	0.86
Ch 41	–67.0	–11.7	8.7	11.5	L-STG (BA 22)	71.6	4.34	0.0002	0.95
Ch 42	–70.0	–40.7	8.7	12.0	L-STG (BA 22)	75.2	3.65	0.0009	0.80
**Japanese (L1) into English (L2)/low familiarity**									
Ch 41	–67.0	–11.7	8.7	11.5	L-STG (BA 22)	71.6	4.67	0.0009	1.02

*SD indicates standard deviation in the spatial estimate; BA indicates Brodmann area; S1 indicates primary somatosensory cortex; DLPFC indicates dorsolateral prefrontal cortex; STG indicates superior temporal gyrus; L and R indicates left and right hemisphere.*

### Cortical Activation Patterns

By integrating the statistical analysis, spatial registration of the channels, and subsequent macroanatomical labeling, the cortical activation patterns observed in the current study are described as below. For the advanced group, there was no significant activation region while translating high-familiarity words from Japanese (L1) into English (L2) and vice versa; however, some regions were activated while translating low-familiarity words. The advanced group elicited greater cerebral hemodynamic responses in one channel registered at Brodmann area 45, the pars triangularis Broca’s area, while translating English (L2) words with low-familiarity into Japanese (L1). On the other hand, the advanced group elicited greater cerebral hemodynamic responses in 12 channels registered at Brodmann areas: 10, the frontopolar area; 22, the superior temporal gyrus; 40, the supramarginal gyrus part of Wernicke’s area; 44, the pars opercularis part of Broca’s area; 45, the pars triangularis Broca’s area; and 46, the dorsolateral prefrontal cortex, while translating Japanese (L1) words with low familiarity into English (L2). For the elementary group, there was significantly or marginally significantly activated regions while translating both from Japanese (L1) into English (L2) and vice versa regardless of word familiarity. When the elementary group translated English (L2) words with high familiarity into Japanese (L1), one channel registered at Brodmann area 22, the superior temporal gyrus, was activated. Also, when the elementary group translated English (L2) words with low familiarity into Japanese (L1) words, one channel registered at Brodmann area 22, the superior temporal gyrus, was marginally significantly activated. For the opposite translation direction, when the elementary group translated Japanese (L1) words with high familiarity into English (L2), four channels registered at Brodmann areas were significantly or marginally significantly activated: 2, the primary somatosensory cortex; 22, the superior temporal gyrus; 43, and the subcentral area. When the elementary group translated Japanese (L1) words with low familiarity into English (L2), one channel registered at Brodmann area 22, the superior temporal gyrus, was significantly activated.

These results show that different brain areas were recruited during word translation between the advanced and the elementary groups. In the advanced group, the frontal area (English-into-Japanese) or the frontal area to the left temporal area (Japanese-into-English) were recruited only during low-familiarity word translation. The results suggest that these regions were involved in the cognitive mechanism with word translation for the advanced group. On the other hand, the results suggest that the activation of the left temporal region was related to translation in the elementary group, regardless of the direction and word familiarity of the translation. A detailed functional description of these areas is given in the “Discussion” section.

### Comparison Between the Advanced and Elementary Groups

We conducted a three-way mixed ANOVA with group (advanced/elementary) as the between-subject factor and direction (English-into-Japanese/Japanese-into-English) and familiarity (high/low) as the within-subject factors to compare brain activations between the advanced and elementary groups (Table 3). Before this, values were averaged between channels corresponding to the same anatomical label for channels activated in a one-sample *t*-test against zero (BA 2: channel 20, BA 10: channels 36, 46, 47, 48, and 49, BA 22: channels 41 and 42, BA 40: channel 10, BA 43: channel 30, BA 44/45: channels 39, 40, and 50, BA 46: channels 25 and 35).

**TABLE 3:** Three-way mixed ANOVA results for behavioral and fNIRS data.

Dependent Value	Reaction times				Accuracy				Activation on BA 2				Activation on BA 10			
				
Factor	*SS*	*MS*	*F*	η*_*p*_^2^*	*SS*	*MS*	*F*	η*_*p*_^2^*	*SS*	*MS*	*F*	η*_*p*_^2^*	*SS*	*MS*	*F*	η_*p*_^2^
Group	12.69	12.69	1.59	0.05	1223.67	1223.67	120.88***	0.77	<0.01	<0.01	0.19	<0.01	0.03	0.03	2.06	0.05
Error-Group	272.05	8.00			374.56	10.12			1.23	0.03		0.57	0.02			
Familiarity	111.28	111.28	49.19***	0.59	1358.82	1358.82	325.32***	0.90	<0.01	<0.001	0.08	<0.01	<0.001	<0.001	<0.01	<0.01
Familiarity × Group	7.33	7.33	3.24	0.09	556.76	556.76	133.30***	0.78	0.01	0.02	9.27**	0.20	0.03	0.03	8.39**	0.18
Error (Familiarity × Group)	76.91	2.26			154.54	4.18			0.08	<0.01		0.16	<0.01			
Direction	52.75	52.75	22.49***	0.40	7.73	7.73	3.75	0.09	<0.01	<0.01	1.06	0.03	0.01	0.01	14.58***	0.28
Direction × Group	0.17	0.17	0.07	<0.01	0.65	0.65	0.32	<0.01	<0.01	<0.001	0.10	<0.01	<0.01	<0.01	7.67**	0.17
Error (Direction × Group)	79.76	2.35			76.24	2.06			0.05	<0.01		0.03	<0.01			
Familiarity × Direction	0.21	0.21	0.13	<0.01	6.89	6.89	4.16*	0.10	<0.01	<0.001	0.52	0.01	<0.001	<0.001	0.02	<0.01
Familiarity × Direction × Group	1.75	1.75	1.03	0.03	31.25	31.25	18.85***	0.34	<0.01	<0.001	0.60	0.02	<0.001	<0.001	0.02	<0.01
Error (Familiarity × Direction × Group)	57.81	1.70			61.34	1.66			0.04	<0.01		0.05	<0.01			

**Dependent Value**	**Activation on BA 22**				**Activation on BA 40**				**Activation on BA 44/45**				**Activation on BA46**			
				
**Factor**	***SS***	***MS***	***F***	**η*_*p*_^2^***	***SS***	***MS***	***F***	**η*_*p*_^2^***	***SS***	***MS***	***F***	**η*_*p*_^2^***	***SS***	***MS***	***F***	**η_*p*_^2^**

Group	0.02	0.02	0.88	0.02	0.04	0.04	2.98	0.07	<0.01	<0.01	0.18	<0.01	0.05	0.05	3.36	0.08
Error-Group	1.06	0.03			0.51	0.01			0.76	0.02			0.56	0.01		
Familiarity	<0.01	<0.01	1.05	0.02	<0.001	<0.001	0.43	0.01	<0.01	<0.01	2.55	0.06	<0.001	<0.001	0.1	<0.01
Familiarity × Group	0.03	0.03	6.19*	0.14	0.01	0.01	6.29*	0.14	0.06	0.06	14.38**	0.27	0.03	0.03	11.01**	0.22
Error (Familiarity × Group)	0.17	<0.01			0.06	<0.01			0.15	<0.01			0.11	<0.01		
Direction	<0.01	<0.01	1.26	0.03	<0.01	<0.01	3.96	0.09	<0.01	<0.01	3.51	0.08	0.02	0.02	8.97**	0.19
Direction × Group	<0.001	<0.001	0.01	<0.001	<0.001	<0.001	0.95	0.02	<0.001	<0.001	0.11	<0.01	<0.01	<0.01	2.54	0.06
Error (Direction × Group)	0.10	<0.01			0.03	<0.001			0.06	<0.01			0.07	<0.01		
Familiarity × Direction	<0.001	<0.001	0.02	<0.001	<0.01	<0.01	1.04	0.03	<0.001	<0.001	0.13	<0.01	<0.001	<0.001	0.02	<0.001
Familiarity × Direction × Group	<0.01	<0.01	5.44	0.01	<0.001	<0.001	0.01	<0.01	<0.01	<0.01	0.48	0.01	<0.001	<0.001	0.05	<0.01
Error (Familiarity × Direction × Group)	0.11	<0.01			0.04	<0.01			0.08	<0.01			0.04	<0.01		

*** Indicates *p* < 0.001, ** indicates *p* < 0.01, * indicates *p* < 0.05. The degrees of freedoms (dfs) for the main effects and interactions of all factors equal 1. The dfs for Errors equal 34 for RT, 37 for ACC, and 38 for activation in brain regions.

In a channel corresponding to the left primary somatosensory cortex (BA 2), there was no significant main effect for group (advanced/elementary), direction (English-into-Japanese/Japanese-into-English), and familiarity (high/low). On the other hand, the interaction between group and familiarity was significant [*F*(1,38) = 9.27, *p* < 0.01, η_*p*_^2^ = 0.20]. The simple main effect of group was larger for low-familiarity words than for high-familiarity words in the advanced group (*p* < 0.05). In channels corresponding to the frontopolar area (BA 10), there was a significant main effect for direction [Japanese-into-English > English-into-Japanese; *F*(1,38) = 14.58, *p* < 0.001, η_*p*_^2^ = 0.27]. The interaction between group and familiarity was significant [*F*(1,38) = 8.39, *p* < 0.01, η_*p*_^2^ = 0.18]. A simple main effect of familiarity was larger for the advanced group than for the elementary group for low-familiarity words (*p* < 0.01). The interaction between group and direction was significant [*F*(1,38) = 7.67, *p* < 0.01, η_*p*_^2^ = 0.17]. The simple main of group effect was larger for the Japanese-into-English direction than for the English-into-Japanese direction in the advanced group (*p* < 0.001). Also, the simple main effect of direction for the advanced group was larger than that for the elementary group in the Japanese-into-English direction (*p* < 0.05). In channels corresponding to the left superior temporal gyrus (BA 22), there was no significant main effect for group, direction, or familiarity. The interaction between group and familiarity was significant [*F*(1,38) = 6.19, *p* < 0.05, η_*p*_^2^ = 0.14]. The simple main effect of group was larger for high-familiarity words than for low-familiarity words in the advanced group (*p* < 0.05). In a channel corresponding to the left Wernicke’s area (BA 40), there was no significant main effect for group, direction, or familiarity. The interaction between group and familiarity was significant [*F*(1,38) = 6.29, *p* < 0.05, η_*p*_^2^ = 0.14]. The simple main effect of group was larger for high-familiarity words than for low-familiarity words in the elementary group (*p* < 0.05). Also, the simple main effect of familiarity was larger for the advanced group than for the elementary group for low-familiarity words (*p* < 0.05). In channels corresponding to the left subcentral area (BA 43), there was no significant main effect for group, direction, or familiarity. Moreover, there were no interactions. In channels corresponding to the left Broca’s area (BA 44/45), there was no significant main effect for group, direction, or familiarity. The interaction between group and familiarity was significant [*F*(1,38) = 6.29, *p* < 0.05, η_*p*_^2^ = 0.14]. The simple main of group effect was larger for high-familiarity words than for low-familiarity words in the elementary group (*p* < 0.05). Also, the simple main effect of familiarity was higher for the advanced group than for the elementary group for low-familiarity words (*p* < 0.05). In channels corresponding to the right DLPFC (BA 46), there was a significant main effect for direction [Japanese-into-English > English-into-Japanese; *F*(1,38) = 8.97, *p* < 0.01, η_*p*_^2^ = 0.19]. The interaction between group and familiarity was significant [*F*(1,38) = 11.01, *p* < 0.01, η_*p*_^2^ = 0.23]. The simple main effect of group was larger for low-familiarity words than for high-familiarity words in the advanced group (*p* < 0.05). The simple main effect of group was larger for high-familiarity words than for low-familiarity words in the elementary group (*p* < 0.05). Also, the simple main effect of familiarity was larger for the advanced group than for the elementary group for low-familiarity words (*p* < 0.01).

These results suggest that language direction and word familiarity had different effects on brain activation between the advanced and elementary groups, with significant interactions in the six regions (the left primary somatosensory cortex: BA 2, the frontopolar area: BA 10, the left superior temporal gyrus: BA 22, the left Wernicke’s area: BA 40, the left Broca’s area: BA 44/45, and the right dorsolateral prefrontal cortex: BA 46). On the other hand, no main effect or interaction was observed for the activation in the left subcentral area (BA 43), which does not support different activation between the two groups.

### Behavioral Data

**FIGURE 6:**
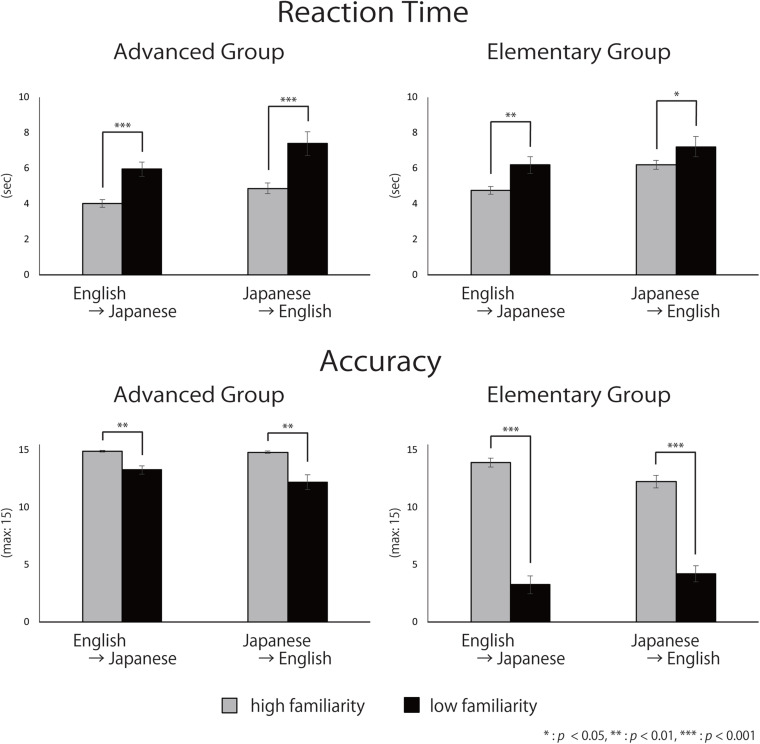
Mean reaction time and accuracy. Error bars indicate standard errors (SE).

The averaged reaction times (RTs) and accuracy for each group are shown in Figure 6. The three-way mixed ANOVA on RTs (Table 3) showed significant main effects of direction [English-into-Japanese < Japanese-into-English; *F*(1,34) = 22.49, *p* < 0.001, η_*p*_^2^ = 0.40] and familiarity [high < low; *F*(1,34) = 49.19, *p* < 0.001, η*_*p*_^2^* = 0.59]. No main effect of group (advanced/elementary) appeared [*F*(1,34) = 1.59, *n.s.*, η_*p*_^2^ = 0.04]. No significant interaction between group and direction [*F*(1,34) = 0.07, *n.s*., η_*p*_^2^ < 0.0001], group and familiarity [*F*(1,34) = 3.24, *n.s*., η_*p*_^2^ = 0.08], direction and familiarity [*F*(1,34) = 0.13, *n.s*., η_*p*_^2^ < 0.0001], or group, direction, and familiarity [*F*(1,34) = 1.03, *n.s.*, η_*p*_^2^ < 0.0001] appeared. The three-way mixed ANOVA on accuracy (Table 3) showed significant main effects of group [advanced > elementary; *F*(1,37) = 120.88, *p* < 0.001, η_*p*_^2^ = 0.77] and familiarity [high > low; *F*(1,37) = 325.32, *p* < 0.001, η_*p*_^2^ = 0.90]. There was no significant main effect of direction [*F*(1,37) = 3.75, *n.s*., η*_*p*_^2^* < 0.0001]. The interaction between group and familiarity was significant [*F*(1,37) = 133.30, *p* < 0.001, η_*p*_^2^ = 0.78]. The simple main effect of familiarity was larger for high-familiarity words than for low-familiarity words in the advanced group (*p* < 0.001). Also, the simple main effect of familiarity was larger for high-familiarity words than for low-familiarity words in the elementary group (*p* < 0.001). The interaction between group, direction, and familiarity was significant [*F*(1,37) = 18.85, *p* < 0.001, η_*p*_^2^ = 0.34]. All simple main effects of familiarity at each level of direction, and all simple main effects of direction at each level of familiarity were larger for the advanced group than for the elementary group (for the high-familiarity words and in the English-into-Japanese direction (*p* < 0.01), for the high-familiarity words and in the Japanese-into-English (*p* < 0.001), for the low-familiarity words and in the English-into-Japanese direction (*p* < 0.001), and for the low-familiarity words and in the Japanese-into-English direction (*p* < 0.001). Since significant three-way interaction was observed for ACC, a two-way interaction contrast was examined for each group. As a result, for the advanced group, the mean value of the contrasts was −0.95 with a standard deviation of 2.72, which was not significant compared to zero [*t*(19) = −1.56, *n.s.*]. On the other hand, for the elementary group, the mean value of the contrasts was 2.63 with a standard deviation of 2.41, which was significantly larger than zero [*t*(18) = 4.76, *p* < 0.001]. Further, probing this interaction contrast in the elementary group, we found that, for high familiarity words, the contrast, English-into-Japanese minus Japanese-into-English, was larger than zero (*p* < 0.001). Conversely, for low familiarity words, the contrast, English-into-Japanese minus Japanese-into-English, was smaller than zero (*p* < 0.05).

To summarize, there were no differences for RTs between the advanced group and the elementary group, whereas there were significant differences for accuracy: the advanced group responded significantly more accurately than did the elementary group. The slower RTs and the lower accuracy for low familiarity words suggest that it is more difficult to translate low-familiarity words than high-familiarity words, regardless of the direction of the translation, for both advanced and elementary groups. However, for the elementary group, there was an interaction between familiarity and direction with the accuracy, suggesting that the elements of the difficulty were different between the advanced and the elementary groups. In addition, regarding ACC, the advanced group exhibited no significant two-way interaction between word familiarity and translation directions. However, the elementary group exhibited a significant two-way interaction. For high-familiarity words, they answered more accurately during English-into-Japanese translation, whereas for low familiarity words, they answered more accurately during Japanese-into-English translation.

## Discussion

We revealed that there were different brain activation patterns while English learners of Japanese translated Japanese (L1) words into English (L2) and vice versa depending on their English proficiency. Specifically, the advanced group elicited greater activation on the left prefrontal cortex around Broca’s area while translating words with low familiarity, but no activation was observed while translating words with high familiarity. On the other hand, the elementary group evoked greater activation on the left temporal area including the superior temporal gyrus (STG) irrespective of word familiarity. These results suggest that different cognitive processes could be involved in word translation depending on English proficiency in Japanese learners of English. Hereafter we will discuss the activation patterns observed in the current study macro-anatomically in reference to previous neuroimaging studies.

### Interpretation of Results

#### Consistent Activation in Broca’s Area (BA 44/45)

In the current study we observed activation in the language-related regions which were also reported in the former studies. First of all, the activation on Broca’s area (BA 44/45) during translation was consistently observed in previous studies (Klein et al., 1995; Rinne et al., 2000; Quaresima et al., 2002; Kovelman et al., 2008a), in which balanced bilinguals translated between languages with close or moderate distances. It has been suggested that the left prefrontal cortex, including the pars opercularis and the pars triangularis of Broca’s area, is related to language comprehension and semantic processing (Devlin et al., 2003). Also, the areas have been revealed as being involved with understanding and retrieval of semantic ambiguity (Rodd et al., 2005). In our study, the advanced group elicited greater activation on Broca’s area when translating words with low familiarity, which should demand higher cognitive loads. It is expected that Broca’s area plays an important role in language processing with high cognitive loads. Considering the previous studies’ results (Klein et al., 1995; Rinne et al., 2000; Quaresima et al., 2002; Kovelman et al., 2008a), it is likely that even balanced bilinguals experience considerable cognitive loads when translating languages with close or moderate LDs. This should be all the more so for advanced English learners translating words in a language with a large LD. For the elementary group, it was difficult to translate words with low familiarity as shown by their low accuracy (Figure 6). Due to the difficulty, they could not translate words with low familiarity and gave up answering correctly. In other words, the elementary group was not able to perform well in word perception itself, which is necessary for word production (Lüders et al., 1991; Indefrey and Levelt, 2000; Indefrey and Levelt, 2004; Hamberger and Cole, 2011). Thus, it is appropriate to interpret that the elementary group did not experience cognitive load or experienced a different kind of cognitive load than the advanced group, thus failing to recruit Broca’s area (BA 44/45).

#### Consistent Activation in the Dorsolateral Prefrontal Cortex (BA 46)

The right dorsolateral prefrontal cortex (R-DLPFC: BA 46) was activated in some previous studies (Klein et al., 1995; Rinne et al., 2000; Kovelman et al., 2008a) and the advanced group in the current study also elicited significant activation in the region while translating Japanese (L1) words with low familiarity into English (L2). The DLPFC is related to verbal working memory (Salmon et al., 1996; Zurowski et al., 2002), which plays an important role in keeping information in mind and processing it simultaneously in a short time (Baddeley, 2003). This region has also been consistently activated during tasks requiring effortful retrieval, maintenance or control of semantic information (Cabeza and Nyberg, 1997). Activation of the right DLPFC was also observed in some previous studies (Klein et al., 1995; Rinne et al., 2000; Kovelman et al., 2008b) focusing on balanced bilinguals. In the present study, the behavioral results showed that the advanced group processed the stimuli more accurately during translation than did the elementary group. Based on the function of the right DLPFC, we considered that such high performance in the advanced group was made possible by their ability to make good use of their verbal working memory. To sum up, the left Broca’s area and the right DLPFC were consistently activated in not only balanced bilinguals whose L1 is closely or moderately related to English, but also in the advanced Japanese learners of English. Therefore, we conclude that these areas are involved with word translation regardless of LDs.

#### Activation Patterns Specifically Obtained in the Current Study

It should be noted that there were several areas that were found to be activated only in the current study. The elementary group elicited greater activation on the left superior temporal gyrus (BA 22) when translating Japanese (L1) into English (L2), and vice versa irrespective of word familiarity. This region was also activated when the advanced group translated Japanese (L1) low familiarity words into English (L2). The STG (BA 22) is reported to play an important role on semantic processing (Warburton et al., 1996) and word retrieval (Hirshorn and Thompson-Schill, 2006). The word translation task in this study required participants to retrieve Japanese (L1) and English (L2) words. However, the cognitive loads with word retrieval depend on the level of automatization of language processing (Segalowitz and Segalowitz, 1993; Ellis, 2002; Suzuki and Sunada, 2018). That is, if word recognition becomes faster and recognition time becomes more stable, then surely there has been a shift toward automatization (Segalowitz et al., 1998). This would imply the fact that with increasing expertise in a second language, learners acquire a richer lexical network for words in L2 (Kroll and De Groot, 2002). In the current study, it appears that language processing of English (L2) for the advanced group was automatized but that for the elementary group was not. This can explain the results that the elementary group elicited significant activation on the STG (BA 22) during both translation directions (Japanese-into-English/English-into-Japanese) irrespective of word familiarity. The STG (BA22) plays an important role in phonological storage within the phonological loop, a subsystem of working memory (Paulesu et al., 1993; Aboitiz et al., 2010; Kekang, 2019). The activation of the STG (BA 22) in the elementary group may reflect that they temporarily stored the stimulus words in the phonological storage before word translation. On the other hand, though we believe that language processing of English (L2) for the advanced group would be rather automatized, translation of unfamiliar Japanese (L1) words into English (L2) would still require high cognitive loads. This might be reflected by the significant activation on the STG (BA 22). This view is also supported by the results of previous behavioral experiments (De Groot and Poot, 1997; Kroll et al., 2010), which showed that cognitive loads when translating L1 into L2 were more burdensome.

Wernicke’s area is involved in various language processes including language comprehension (Ardila et al., 2016). In particular, the left supramarginal gyrus part of Wernicke’s area (BA 40) is related to word recognition (DeWitt and Rauschecker, 2013). In the current study, the advanced group elicited significant activation in BA 40 when translating unfamiliar Japanese (L1) words into English (L2). The cognitive mechanisms required in language translation are considered to be different depending on the differences in language direction. That is, L1-into-L2 translation has stronger lexical and semantic demands associated with processing input in L2 as opposed to L1 compared to L2-into-L1 translation (Christoffels et al., 2013). Generally behavioral performance is typically worse for L1-into-L2 translation than L2-into-L1 (Kroll and Stewart, 1994; De Groot and Poot, 1997; Kroll et al., 2010). In the present study, a main effect of language direction was observed for RT during translation. Therefore, the higher lexical and semantic demands associated with the processing of input in L2 may have elicited the activation of Wernicke’s area (BA 40) in the advanced group.

The activation in the STG mainly found in the elementary group and the activation in Wernicke’s area specifically found in the advanced group are consistent with the existing dual-route process model of second language acquisition (Kroll and Stewart, 1994; Kroll and de Groot, 1997; Duyck and Brysbaert, 2008). In the elementary group, semantic route processing seems to have been dominant, regardless of the translation direction and word familiarity. In the elementary group, the word concept was processed with the semantic route because a sufficient amount of vocabulary was not stored. Accordingly, the semantic route may have elicited activation of the STG, but not Wernicke’s area, associated with vocabulary storage (Paulesu et al., 1993; Aboitiz et al., 2010; Kekang, 2019). On the other hand, in the advanced group, because of the relatively rich vocabulary storage, lexical route processing (Kroll and Stewart, 1994; Kroll and de Groot, 1997; Duyck and Brysbaert, 2008) for English (L2)-into-Japanese (L1) translation similar to bilingual second language processing (e.g., Green, 1998) may have taken place, resulting in activation in Wernicke’s area.

The frontopolar area (BA 10) has been reported to serve a function in the processing of cognitive branching (Koechlin and Hyafil, 2007), in which we maintain in working memory a primary goal, while at the same time processing tasks related to a secondary goal (Ramnani and Owen, 2004). This region was activated when the advanced group translated Japanese (L1) words with low familiarity into English (L2). As in the case of BA 22, we suggest that BA 10 activation is another indicator of the large cognitive loads that advanced English learners have when translating unfamiliar L1 words into L2.

### Limitations and Perspectives of This Experiment

Although we affirmed the brain activation patterns for Japanese learners of English during word translation with a large LD, there are some limitations as to the investigation of the mechanism of Japanese learners acquiring English. First, our study did not make clear how brain activation patterns for the elementary group change into those for the advanced group. It is unclear whether it would be continuous or discrete. For the future, examining brain activation patterns for Japanese learners of English with an intermediate level would allow us to clarify the transition of cognitive mechanisms with increasing English levels. Alternatively, longitudinal studies on how elementary learners become advanced would provide clearer evidence for the differential activation. Second, we did not investigate brain activation patterns for Japanese learners of English who are balanced bilinguals. Thus, cortical activation patterns for Japanese learners who completely acquire English remains uncertain. In our study, we recruited an advanced group whose TOEIC^®^ scores were over the average score of Japanese learners. However, there are few Japanese learners in the advanced group who are considered balanced bilinguals. Therefore, to fully understand the mechanism of acquiring English by Japanese learners with a large LD, we need to examine brain activation patterns on balanced bilinguals whose L1 is Japanese and L2 is English. Finally, we measured only the frontal and temporal regions with multichannel fNIRS due to the inherent spatial limitations of the fNIRS setup. With this limitation in mind, we carefully selected the measurement areas based on previous results (e.g., Klein et al., 1995; Price et al., 1999; Quaresima et al., 2002) related with language translation. Though we have these limitations to consider, we present significant findings that brain activation patterns for Japanese learners of English vary depending on the level of acquired English and cognitive loads of translation tasks. This study provides the first evidence revealing the cognitive mechanisms during word translation between languages at a large LD from a functional neuroimaging perspective. Furthermore, our study may serve to provide an effective cognitive strategy for Japanese learners of English at the elementary level. Our results show that cortical activation on the left STG was observed for the elementary group, while Wernicke’s area was activated for the advanced group. These results may reflect whether the semantic or lexical route was dominant when English learners processed words such as during translation. However, since our data were not longitudinal and we have yet to provide definitive evidence for proving this hypothesis, we still need to verify that the differences in performance and cortical activation between the advanced and elementary groups reflect the improvement of English proficiency as a second language. There has been a lot of discussion about cognitive strategies in language acquisition. The depth of lexical knowledge is related to word perception (Ouellette, 2006). For processing with the lexical route, it is necessary to improve the mental lexicon for the second language and to increase accessibility to it (Talamas et al., 1999; Kroll and Tokowicz, 2001; Ouellette, 2006). It will be interesting to incorporate these plausible factors in future studies to examine the relationship between cortical activation in Japanese learners of English at the elementary level during word translation and cognitive strategies. Together with the current findings, such an integrated examination may provide insight into effective cognitive strategies for second language acquisition.

## Data Availability Statement

The raw data supporting the conclusions of this article will be made available by the authors, without undue reservation.

## Ethics Statement

The studies involving human participants were reviewed and approved by Ethics Committee of Chuo University. The patients/participants provided their written informed consent to participate in this study.

## Author Contributions

KS and ID devised the idea of this study. KS and TaT selected and created the experimental stimuli. KS, KN, TaT, KO, and ToT prepared the fNIRS measurements. KN, TaT, KO, and ToT collected and performed the data analyses. KN created the experimental programming. YK reviewed the statistical procedures. KS, KN, TaT, and ID wrote the manuscript. All the authors have read the final version of the manuscript and agreed to its publication.

## Conflict of Interest

The authors declare that the research was conducted in the absence of any commercial or financial relationships that could be construed as a potential conflict of interest.

## References

[B1] Aboitiz F. Aboitiz S. García R. R. (2010). The phonological loop: a key innovation in human evolution. *Curr. Anthropol.* 51 55–65.

[B2] Agency for Cultural Affairs (2010). *Jyoyo Kanji List (in Japanese).* Available online at: https://www.bunka.go.jp/kokugo_nihongo/sisaku/joho/joho/kijun/naikaku/pdf/joyokanjihyo_20101130.pdf. [Acccessed on August 7, 2020]

[B3] Amano S. Kondo T. (1998). “Estimation of mental lexicon size with word familiarity database,” in *Proceedings of the Fifth International Conference on Spoken Language Processing*, (Sydney, NSW).

[B4] Ardila A. Bernal B. Rosselli M. (2016). How localized are language brain areas? a review of brodmann areas involvement in oral language. *Arch. Clin. Neuropsychol.* 31 112–122. 10.1093/arclin/acv081 26663825

[B5] Baddeley A. (2003). Working memory and language: an overview. *J. Commun. Disord.* 36 189–208. 10.1016/s0021-9924(03)00019-4 12742667

[B6] Brainard D. H. (1997). The psychophysics toolbox. *Spat. Vis.* 10 433–436. 10.1163/156856897x00357 9176952

[B7] British National, and Corpus. (2007). *British National Corpus.* Available online at: https://www.english-corpora.org/bnc/. [Acccessed on August 7, 2020]

[B8] Cabeza R. Nyberg L. (1997). Imaging cognition: an empirical review of PET studies with normal subjects. *J. Cogn. Neurosci.* 9 1–26. 10.1162/jocn.1997.9.1.1 23968177

[B9] Cambridge University, and Press. (2020). *Cambridge Dictionary.* Available online at: https://dictionary.cambridge.org/ja/. [Acccessed on August 7, 2020].

[B10] Chiswick B. R. Miller P. W. (2005). Linguistic distance: a quantitative measure of the distance between english and other languages. *J. Multilingual Multicult. Dev.* 26 1–11. 10.1080/14790710508668395

[B11] Christoffels I. K. Ganushchak L. Koester D. (2013). Language conflict in translation: an ERP study of translation production. *J. Cogn. Psychol.* 25 646–664. 10.1080/20445911.2013.821127

[B12] Crystal D. (2008). Two thousand million? *English Today* 24 3–6. 10.1017/s0266078408000023

[B13] Crystal D. (2012). *English as a Global Language*, 2nd Edn. Cambridge: Cambridge University Press.

[B14] Cui X. Bray S. Reiss A. L. (2010). Functional near infrared spectroscopy (NIRS) signal improvement based on negative correlation between oxygenated and deoxygenated hemoglobin dynamics. *Neuroimage* 49 3039–3046. 10.1016/j.neuroimage.2009.11.050 19945536 PMC2818571

[B15] Damasio H. Grabowski T. J. Tranel D. Hichwa R. D. Damasio A. R. (1996). A neural basis for lexical retrieval. *Nature* 380 499–505. 10.1038/380499a0 8606767

[B16] De Groot A. M. Poot R. (1997). Word translation at three levels of proficiency in a second language: the ubiquitous involvement of conceptual memory. *Lang. Learn.* 47 215–264. 10.1111/0023-8333.71997007

[B17] Delpy D. T. Cope M. van der Zee P. Arridge S. Wray S. Wyatt J. (1988). Estimation of optical pathlength through tissue from direct time of flight measurement. *Phys. Med. Biol.* 33 1433–1442. 10.1088/0031-9155/33/12/008 3237772

[B18] Devlin J. T. Matthews P. M. Rushworth M. F. (2003). Semantic processing in the left inferior prefrontal cortex: a combined functional magnetic resonance imaging and transcranial magnetic stimulation study. *J. Cogn. Neurosci.* 15 71–84. 10.1162/089892903321107837 12590844

[B19] DeWitt I. Rauschecker J. P. (2013). Wernicke’s area revisited: parallel streams and word processing. *Brain Lang.* 127 181–191. 10.1016/j.bandl.2013.09.014 24404576 PMC4098851

[B20] Duyck W. Brysbaert M. (2008). Semantic access in number word translation: the role of crosslingual lexical similarity. *Exp. Psychol.* 55 102–112. 10.1027/1618-3169.55.2.102 18444520

[B21] Educational Testing and Service (2020a). *Test of English as a Foreign Language, Internet-based Test.* Available online at: https://www.ets.org/s/toefl/pdf/94227_unlweb.pdf. [Acccessed on August 7, 2020]

[B22] Educational Testing and Service (2020b). *Test of English for International Communication.* Available online at: https://www.ets.org/s/toeic/pdf/2018-report-on-test-takers-worldwide.pdf. [Acccessed on August 7, 2020]

[B23] Ellis N. C. (2002). Frequency effects in language processing: a review with implications for theories of implicit and explicit language acquisition. *Stud. Second Lang. Acquisition* 24 143–188. 10.1017/s0272263102002024

[B24] Friston K. J. Fletcher P. Josephs O. Holmes A. Rugg M. Turner R. (1998). Event-related fMRI: characterizing differential responses. *Neuroimage* 7 30–40. 10.1006/nimg.1997.0306 9500830

[B25] Green D. W. (1986). Control, activation, and resource: a framework and a model for the control of speech in bilinguals. *Brain Lang.* 27 210–223. 10.1016/0093-934x(86)90016-7 2420411

[B26] Green D. W. (1998). Mental control of the bilingual lexico-semantic system. *Bilingual. Lang. Cogn.* 1 67–81. 10.1017/s1366728998000133

[B27] Grosjean F. (1997). “Processing mixed language: issues, findings and models,” in *Tutorials In Bilingualism: Psycholinguistic Perspectives*, eds de Groot A. M. B. Kroll J. F. (Mahwah, NJ: Lawrence Erlbaum Publishers). 225–254.

[B28] Hamberger M. J. Cole J. (2011). Language organization and reorganization in epilepsy. *Neuropsychol. Rev.* 21 240–251. 10.1007/s11065-011-9180-z 21842185 PMC3193181

[B29] Hervais-Adelman A. Moser-Mercer B. Michel C. M. Golestani N. (2015). fMRI of simultaneous interpretation reveals the neural basis of extreme language control. *Cereb. Cortex* 25 4727–4739. 10.1093/cercor/bhu158 25037924

[B30] Hirshorn E. A. Thompson-Schill S. L. (2006). Role of the left inferior frontal gyrus in covert word retrieval: neural correlates of switching during verbal fluency. *Neuropsychologia* 44 2547–2557. 10.1016/j.neuropsychologia.2006.03.035 16725162

[B31] Homae F. Watanabe H. Nakano T. Taga G. (2007). Prosodic processing in the developing brain. *Neurosci. Res.* 59 29–39. 10.1016/j.neures.2007.05.005 17590470

[B32] Homae F. Watanabe H. Nakano T. Taga G. (2011). Large-scale brain networks underlying language acquisition in early infancy. *Front. Psychol.* 2:93. 10.3389/fpsyg.2011.00093 21687461 PMC3110337

[B33] Huppert T. J. Hoge R. D. Diamond S. G. Franceschini M. A. Boas D. A. (2006). A temporal comparison of BOLD, ASL, and NIRS hemodynamic responses to motor stimuli in adult humans. *Neuroimage* 29 368–382. 10.1016/j.neuroimage.2005.08.065 16303317 PMC2692693

[B34] IELTS Partners (2020). *International English Language Testing System.* Available online at: https://www.ielts.org/policy/copyright-notice. [Acccessed on August 7, 2020]

[B35] Indefrey P. Levelt W. J. (2000). “The neural correlates of language production,” in *The New cognitive Neurosciences*, 2nd Edn, ed. Gazzaniga M. S. (Cambridge, MA: MIT press), 845–865.

[B36] Indefrey P. Levelt W. J. M. (2004). The spatial and temporal signatures of word production components. *Cognition* 92 101–144. 10.1016/j.cognition.2002.06.001 15037128

[B37] Jang K.-E. Tak S. Jung J. Jang J. Jeong Y. Ye Y. C. (2009). Wavelet minimum description length detrending for near-infrared spectroscopy. *J. Biomed. Opt.* 14:034004. 10.1117/1.3127204 19566297

[B38] Jeong H. Sugiura M. Sassa Y. Yokoyama S. Horie K. Sato S. (2007). Cross-linguistic influence on brain activation during second language processing: an fMRI study. *Bilingualism* 10 175–187. 10.1017/s1366728907002921

[B39] Jescheniak J. D. Levelt W. J. (1994). Word frequency effects in speech production: retrieval of syntactic information and of phonological form. *J. Exp. Psychol. Learn. Memory Cogn.* 20 824–843. 10.1037/0278-7393.20.4.824

[B40] Jurcak V. Tsuzuki D. Dan I. (2007). 10/20, 10/10, and 10/5 systems revisited: their validity as relative head-surface-based positioning systems. *Neuroimage* 34 1600–1611. 10.1016/j.neuroimage.2006.09.024 17207640

[B41] Kawabata Duncan K. Tokuda T. Sato C. Tagai K. Dan I. (2019). Willingness-to-pay-associated right prefrontal activation during a single, real use of cosmetics as revealed by functional near-infrared spectroscopy. *Front. Hum. Neurosci.* 13:16. 10.3389/fnhum.2019.00016 30778292 PMC6369365

[B42] Kekang H. (2019). *Semantic Perception Theory: A New Theory on Children’s Language Development.* Singapor: Springer Verlag, 26–36.

[B43] Kirkpatrick A. (2012). English as an Asian Lingua Franca: the ‘Lingua Franca approach’ and implications for language education policy. *J. English Lingua Franca* 1 121–139.

[B44] Klein D. Milner B. Zatorre R. J. Meyer E. Evans A. C. (1995). The neural substrates underlying word generation: a bilingual functional-imaging study. *Proc. Natl. Acad. Sci. U S A.* 92 2899–2903. 10.1073/pnas.92.7.2899 7708745 PMC42326

[B45] Kleiner M. Brainard D. Pelli D. (2007). What’s new in psychtoolbox-3? *Perception* 36 1–16.

[B46] Klem G. H. Luders H. O. Jasper H. H. Elger C. (1999). The ten-twenty electrode system of the international federation. the international federation of clinical neurophysiology. *Electroencephalogr. Clin. Neurophysiol. Suppl.* 52 3–6. 10590970

[B47] Koechlin E. Hyafil A. (2007). Anterior prefrontal function and the limits of human decision-making. *Science (New York, N.Y.)* 318 594–598. 10.1126/science.1142995 17962551

[B48] Konishi T. Minamide K. (2001). *Taishukan’s Genius Unabridged English-Japanese Dictionary.* Tokyo: Taishukan.

[B49] Kovelman I. Baker S. A. Petitto L.-A. (2008a). Bilingual and monolingual brains compared: a functional magnetic resonance imaging investigation of syntactic processing and a possible “neural signature” of bilingualism. *J. Cogn. Neurosci.* 20 153–169. 10.1162/jocn.2008.20011 17919083 PMC2643466

[B50] Kovelman I. Shalinsky M. H. Berens M. S. Petitto L. A. (2008b). Shining new light on the brain’s “bilingual signature”: a functional near infrared spectroscopy investigation of semantic processing. *Neuroimage* 39 1457–1471. 10.1016/j.neuroimage.2007.10.017 18054251 PMC2249758

[B51] Kroll J. F. Stewart E. (1994). Category interference in translation and picture naming: evidence for asymmetric connections between bilingual memory representations. *J. Memory Lang.* 33 149–174. 10.1006/jmla.1994.1008

[B52] Kroll J. F. Tokowicz N. (2001). The development of conceptual representation for words in a second language. *One Mind Two Lang. Bilingual Lang. Process.* 2 49–71.

[B53] Kroll J. F. Van Hell J. G. Tokowicz N. Green D. W. (2010). The revised hierarchical model: a critical review and assessment. *Bilingual. Lang. Cogn.* 13 373–381. 10.1017/s136672891000009x 20676387 PMC2910435

[B54] Kroll J. F. de Groot A. M. B. (1997). “Lexical and conceptual memory in the bilingual: Mapping form to meaning in two languages,” in *Tutorials In Bilingualism: Psycholinguistic Perspectives.* eds de Groot A. M. B. Kroll J. F. (Mahwah, NJ: Lawrence Erlbaum Publishers), 201–224.

[B55] Kroll J. F. De Groot A. M. (2002). Mapping form to meaning in two languages. *Psychol. Crit. Concepts Psychol.* 2 203–234.

[B56] Kubozono H. (1989). The mora and syllable structure in Japanese: evidence from speech errors. *Lang. Speech* 32 249–278. 10.1177/002383098903200304

[B57] Laine M. Rinne J. O. Krause B. J. Teräs M. Sipilä H. (1999). Left hemisphere activation during processing of morphologically complex word forms in adults. *Neurosci. Lett.* 271 85–88. 10.1016/S0304-3940(99)00527-523 10477108

[B58] Laufer B. Nation P. (1999). A vocabulary-size test of controlled productive ability. *Lang. Test.* 16 33–51. 10.1177/026553229901600103

[B59] Lawson A. J. (2008). Testing the TOEIC: Practicality, Reliability and Validity in the Test of English for International Communication. Available online at: http://www.cels.bham.ac.uk/resources/essays/andy-lawson-testing.pdf (accessed November 21, 2020).

[B60] Lehtonen M. H. Laine M. Niemi J. Thomsen T. Vorobyev V. A. Hugdahl K. (2005). Brain correlates of sentence translation in Finnish–Norwegian bilinguals. *NeuroReport* 16 607–610. 10.1097/00001756-200504250-00018 15812317

[B61] Lei M. Miyoshi T. Niwa Y. Dan I. Sato H. (2018). Comprehension-dependent cortical activation during speech comprehension tasks with multiple languages: functional near-infrared spectroscopy study. *Japanese Psychol. Res.* 60 300–310. 10.1111/jpr.12218

[B62] Lüders H. Lesser R. P. Hahn J. Dinner D. Morris H. Wyllie E. (1991). Basal temporal language area. *Brain* 114 743–754. 10.1093/brain/114.2.743 2043946

[B63] Maki A. Yamashita Y. Ito Y. Watanabe E. Mayanagi Y. Koizumi H. (1995). Spatial and temporal analysis of human motor activity using noninvasive NIR topography. *Med. Phys.* 22 1997–2005. 10.1118/1.597496 8746704

[B64] May L. Gervain J. Carreiras M. Werker J. F. (2018). The specificity of the neural response to speech at birth. *Dev. Sci.* 21:e12564. 10.1111/desc.12564 28503845

[B65] Mayer K. M. Yildiz I. B. Macedonia M. von Kriegstein K. (2015). Visual and motor cortices differentially support the translation of foreign language words. *Curr. Biol.* 25 530–535. 10.1016/j.cub.2014.11.068 25660537

[B66] Meyer A. S. Huettig F. Levelt W. J. (2016). Same, different, or closely related: what is the relationship between language production and comprehension? *J. Memory Lang.* 89 1–7. 10.1016/j.jml.2016.03.002

[B67] Minagawa-Kawai Y. Mori K. Furuya I. Hayashi R. Sato Y. (2002). Assessing cerebral representations of short and long vowel categories by NIRS. *Neuroreport* 13 581–584. 10.1097/00001756-200204160-00009 11973450

[B68] Minagawa-Kawai Y. Mori K. Naoi N. Kojima S. (2007). Neural attunement processes in infants during the acquisition of a language-specific phonemic contrast. *J. Neurosci.* 27 315–321. 10.1523/jneurosci.1984-06.2007 17215392 PMC6672067

[B69] Minagawa-Kawai Y. Mori K. Sato Y. Koizumi T. (2004). Differential cortical responses in second language learners to different vowel contrasts. *NeuroReport* 15 899–903. 10.1097/00001756-200404090-00033 15073539

[B70] Niioka K. Uga M. Nagata T. Tokuda T. Dan I. Ochi K. (2018). Cerebral hemodynamic response during concealment of information about a mock crime: application of a general linear model with an adaptive hemodynamic response function. *Japanese Psychol. Res.* 60 311–326. 10.1111/jpr.12194

[B71] Nomura K. Hanamoto K. Hayashi R. (2016). *OLEX English Japanese Dictionary.* Tokyo: Obunsha.

[B72] Obrig H. Mock J. Stephan F. Richter M. Vignotto M. Rossi S. (2017). Impact of associative word learning on phonotactic processing in 6-month-old infants: a combined EEG and fNIRS study. *Dev. Cogn. Neurosci.* 25 185–197. 10.1016/j.dcn.2016.09.001 27692617 PMC6987754

[B73] Obrig H. Rossi S. Telkemeyer S. Wartenburger I. (2010). From acoustic segmentation to language processing: evidence from optical imaging. *Front. Neuroenerget.* 2:13. 10.3389/fnene.2010.00013 20725516 PMC2912026

[B74] Okamoto M. Dan I. (2005). Automated cortical projection of head-surface locations for transcranial functional brain mapping. *Neuroimage* 26 18–28. 10.1016/j.neuroimage.2005.01.018 15862201

[B75] Oldfield R. C. (1971). The assessment and analysis of handedness: the Edinburgh inventory. *Neuropsychologia* 9 97–113. 10.1016/0028-3932(71)90067-4 5146491

[B76] Ong J. Zhang L. J. (2010). Effects of task complexity on the fluency and lexical complexity in EFL students’ argumentative writing. *J. Second Lang. Writing* 19 218–233. 10.1016/j.jslw.2010.10.003

[B77] Ouellette G. P. (2006). What’s meaning got to do with it: the role of vocabulary in word reading and reading comprehension. *J. Educ. Psychol.* 98 554–566. 10.1037/0022-0663.98.3.554

[B78] Paradis M. (1997). “The cognitive neuropsychology of bilingualism,” in *Tutorials in Bilingualism: Psycholinguistic Perspectives*, eds de Groot A. M. B. Kroll J. F. (New Jersey, NJ: Lawrence Erlbaum Associates Publishers), 331–354.

[B79] Patterson K. Shewell C. Coltheart M. Sartori G. Job R. (1987). “Speak and spell: dissociations and word-class effects,” in *The Cognitive Neuropsychology of Language*, eds Coltheart M. Sartori G. Job R. (New Jersey, NJ: Lawrence Erlbaum Associates, Inc), 273–294.

[B80] Paulesu E. Frith C. D. Frackowiak R. S. (1993). The neural correlates of the verbal component of working memory. *Nature* 362 342–345. 10.1038/362342a0 8455719

[B81] Pelli D. G. (1997). The VideoToolbox software for visual psychophysics: transforming numbers into movies. *Spatial Vis.* 10 437–442. 10.1163/156856897x00366 9176953

[B82] Price C. J. Green D. W. Von Studnitz R. (1999). A functional imaging study of translation and language switching. *Brain* 122 2221–2235. 10.1093/brain/122.12.2221 10581218

[B83] Price C. J. Moore C. J. Humphreys G. W. Wise R. J. (1997). Segregating semantic from phonological processes during reading. *J. Cogn. Neurosci.* 9 727–733. 10.1162/jocn.1997.9.6.727 23964595

[B84] Quaresima V. Ferrari M. van der Sluijs M. C. Menssen J. Colier W. N. (2002). Lateral frontal cortex oxygenation changes during translation and language switching revealed by non-invasive near-infrared multi-point measurements. *Brain Res. Bull.* 59 235–243. 10.1016/s0361-9230(02)00871-7 12431754

[B85] Ramnani N. Owen A. (2004). Anterior prefrontal cortex: insights into function from anatomy and neuroimaging. *Nat. Rev. Neurosci.* 5 184–194. 10.1038/nrn1343 14976518

[B86] Rinne J. O. Tommola J. Laine M. Krause B. J. Schmidt D. Kaasinen V. (2000). The translating brain: cerebral activation patterns during simultaneous interpreting. *Neurosci. Lett.* 294 85–88. 10.1016/s0304-3940(00)01540-8 11058793

[B87] Rodd J. M. Davis M. H. Johnsrude I. S. (2005). The neural mechanisms of speech comprehension: fMRI studies of semantic ambiguity. *Cereb. Cortex* 15 1261–1269. 10.1093/cercor/bhi009 15635062

[B88] Rorden C. Brett M. (2000). Stereotaxic display of brain lesions. *Behav. Neurol.* 12 191–200. 10.1155/2000/421719 11568431

[B89] Salmon E. Van der Linden M. Collette F. Delfiore G. Maquet P. Degueldre C. (1996). Regional brain activity during working memory tasks. *Brain* 119 1617–1625. 10.1093/brain/119.5.1617 8931584

[B90] Segalowitz N. S. Segalowitz S. J. (1993). Skilled performance, practice, and the differentiation of speed-up from automatization effects: evidence from second language word recognition. *Appl. Psychol.* 14 369–385. 10.1017/s0142716400010845

[B91] Segalowitz S. J. Segalowitz N. S. Wood A. G. (1998). Assessing the development of automaticity in second language word recognition. *Appl. Psychol.* 19 53–67. 10.1017/s0142716400010572

[B92] Seghier M. L. Price C. J. (2012). Functional heterogeneity within the default network during semantic processing and speech production. *Frontiers in psychology* 3:281. 10.3389/fpsyg.2012.00281 22905029 PMC3417693

[B93] Seidlhofer B. (2005). English as a lingua franca. *ELT J.* 59 339–341.

[B94] Singh A. K. Okamoto M. Dan H. Jurcak V. Dan I. (2005). Spatial registration of multichannel multi-subject fNIRS data to MNI space without MRI. *Neuroimage* 27 842–851. 10.1016/j.neuroimage.2005.05.019 15979346

[B95] Sugiura L. Hata M. Matsuba-Kurita H. Uga M. Tsuzuki D. Dan I. (2018). Explicit performance in girls and implicit processing in boys: a simultaneous fNIRS–ERP study on second language syntactic learning in young adolescents. *Front. Hum. Neurosci.* 12:62. 10.3389/fnhum.2018.00062 29568265 PMC5853835

[B96] Suzuki Y. Sunada M. (2018). Automatization in second language sentence processing: relationship between elicited imitation and maze tasks. *Bilingual. Lang. Cogn.* 21 32–46. 10.1017/s1366728916000857

[B97] Talamas A. Kroll J. F. Dufour R. (1999). From form to meaning: stages in the acquisition of second-language vocabulary. *Bilingual. Lang. Cogn.* 2 45–58. 10.1017/s1366728999000140

[B98] Tsuzuki D. Dan I. (2014). Spatial registration for functional near-infrared spectroscopy: from channel position on the scalp to cortical location in individual and group analyses. *Neuroimage* 85 92–103. 10.1016/j.neuroimage.2013.07.025 23891905

[B99] Tsuzuki D. Jurcak V. Singh A. K. Okamoto M. Watanabe E. Dan I. (2007). Virtual spatial registration of stand-alone fNIRS data to MNI space. *Neuroimage* 34 1506–1518. 10.1016/j.neuroimage.2006.10.043 17207638

[B100] Vandenberghe R. Price C. Wise R. Josephs O. Frackowiak R. S. (1996). Functional anatomy of a common semantic system for words and pictures. *Nature* 383 254–256. 10.1038/383254a0 8805700

[B101] Votaw M. C. (1992). A functional view of bilingual lexicosemantic organization. *Adv. Psychol.* 89 299–321. 10.1016/s0166-4115(08)61502-2

[B102] Warburton E. Wise R. J. Price C. J. Weiller C. Hadar U. Ramsay S. (1996). Noun and verb retrieval by normal subjects studies with PET. *Brain* 119 159–179. 10.1093/brain/119.1.159 8624678

[B103] Yokokawa H. Satoi H. Shimamoto T. Tanimura M. Hirai A. Yabuuchi S. (2007). A comparison of differences between auditory and visual presentations of english word familiarity ratings among japanese efl learners. *Lang. Educ. Technol.* 44 205–214.

[B104] Zheng B. Báez S. Su L. Xiang X. Weis S. Ibáñez A. (2020). Semantic and attentional networks in bilingual processing: fMRI connectivity signatures of translation directionality. *Brain Cogn.* 143:105584. 10.1016/j.bandc.2020.105584 32485460 PMC7933822

[B105] Zurowski B. Gostomzyk J. Grön G. Weller R. Schirrmeister H. Neumeier B. (2002). Dissociating a common working memory network from different neural substrates of phonological and spatial stimulus processing. *NeuroImage* 15 45–57. 10.1006/nimg.2001.0968 11771973

